# Toward better home visits: a mixed-methods study identifying disparities in early childhood program delivery to promote health equity

**DOI:** 10.1186/s12913-026-14092-2

**Published:** 2026-02-02

**Authors:** Madelene Barboza, Mattias Wennergren, Åsa Nilses, Susanne Bernhardsson, Julie S. Lundgren

**Affiliations:** 1 Equity and Health Policy Research Group, Department of Global Public Health, Karolinska Institutet, Stockholm, Sweden; 2 General Practice / Family Medicine, School of Public Health and Community Medicine, Institute of Medicine, Sahlgrenska Academy, University of Gothenburg, Box 454, 405 30, Gothenburg, Sweden; 3 Research, Education, Development and Innovation Primary Health Care, Gothenburg, Region Västra Götaland, Sweden; 4 Center for Progress in Children’s Mental Health, Gothenburg, Region Västra Götaland Sweden; 5 University of Gothenburg, Sahlgrenska Academy, Institute of Neuroscience and Physiology, Department of Health and Rehabilitation, Gothenburg, Sweden

**Keywords:** Extended home visits, Implementation fidelity, Early childhood development, Health equity, Mixed-methods research, Nurturing care

## Abstract

**Background:**

Research and policy developments in the area of early childhood development and health equity have led to an increase in parent support interventions. An extended home visiting program within Child Health Services in collaboration with social services was developed to improve health equity among children in socioeconomically deprived areas as an early, trust-based, and proportionate universalism intervention. To ensure that evidence-based program models are replicated and implemented successfully, it is essential to monitor fidelity to core components during program delivery. This study aims to investigate the delivery of home visits during the scale-up of an early childhood home visiting model in socio-economically disadvantaged areas in Gothenburg, Sweden, with particular focus on health equity.

**Methods:**

A convergent mixed-methods approach was applied, using data from fidelity monitoring collected through a questionnaire filled out after each visit by the nurses and social workers who provided the home visits. Initial directed content analysis with pre-determined categories was carried out. The qualitative findings informed a subsequent quantitative analysis, which included non-parametric testing to compare program delivery based on variation in home visit characteristics.

**Results:**

The reported content of home visits was very similar to the original program core components. Professionals indicated high levels of satisfaction with their ability to implement the program model, successfully working in professional teams, and establishing an alliance with families. At the same time, communication and parents’ participation were noted as challenging aspects. Disparities in program delivery were found between visits with and without an interpreter present, as well as visits with one or both parents present.

**Conclusions:**

The findings suggest successful replication of the program model during scale-up. However, the disparities observed in staff reports on program delivery represent a threat to the principles of proportionate universalism and could potentially increase inequities in the conditions for optimal child development. It is therefore essential to develop strategies to overcome language barriers, to promote fathers’ continued engagement, and to ensure equitable program delivery.

**Trial registration:**

The study was retrospectively registered on 19/09/2024 in the ISRCTN registry (ISRCTN19253469).

**Supplementary Information:**

The online version contains supplementary material available at 10.1186/s12913-026-14092-2.

## Background

During the last three decades, research and policy in the field of early childhood development (ECD) have expanded the focus that previously lay on survival to embrace the right of all children to develop to their full potential [1–3]. This is clearly presented in the World Health Organization’s framework of nurturing care, which includes a comprehensive strategy and a set of guidelines developed for policymakers to design relevant and effective ECD programs [4]. The framework presents the necessary conditions to ensure healthy ECD from a holistic perspective that involves caregivers, families, services and policies. Nurturing care includes five components: good health; adequate nutrition; responsive caregiving; opportunities for early learning; and security and safety. The five components targets the interaction between caregiver and child, while services, programs and policies should create enabling environments in terms of support to the families. The health sector is central, but multiple sectors and cross-sectoral collaborations are also necessary [1, 2, 4].

The importance of healthy ECD has also been shown to be integral to attaining health equity in the past decades. An important example was the proclamation of early childhood as one of the crucial focus areas for the promotion of health equity in the reports of the World Health Organization’s Commission on the Social Determinants of Health [5, 6]. The Commission also issued the policy recommendation of proportionate universalism as a mean to level the social gradient where health status in populations gradually worsens when moving down the socioeconomic ladder. The policy combines the universalist principle with the principle of targeting. In practice, this means guaranteeing universal access to services and resources for the whole population, while offering a higher dose of support to meet the needs of higher risk groups [5, 7, 8].

The development of nurturing care and proportionate universalism policies has been influential in initiating the development of an array of programs to promote equitable ECD. However, translating theory to practice through implementing interventions has been met with some barriers. For example, it has been proven challenging to scale-up smaller ECD interventions to promote nurturing care while maintaining fidelity and favorable outcomes [9–11]. Scaling-up a program model requires effective strategies for how to integrate the intervention into existing service systems and institutional structures (9, 11). It is also essential to ensure the implementation of the original core components with good fidelity while still allowing for necessary adaptation to the new context [12]. To advance the field of ECD interventions at scale it is also necessary to improve monitoring and evaluation of the implementation processes [13, 14]. Regarding proportionate universalism, it has been pointed out that the theoretical concept is too vague when it comes to guidance for practical implementation [15–17]. A scoping review on interventions using proportionate universalism has further confirmed that most of them, despite focusing on health inequities, did not include the aim of lowering the social-gradient, which would need to consider the gradual worsening of health status within the whole population and not only the most vulnerable groups [18].

Within the international context, Sweden has a comparably well-functioning welfare system based on tax-funded resource allocation. For children, preventive health services are delivered through the universal child health care (CHC) program. The program is free of charge and while voluntary, about 99% of all children attend the program, delivered by pediatric specialty nurses, that includes health monitoring, vaccinations and parent support [19, 20]. The CHC is organized under universal primary health care and is financed by the healthcare centre’s global budget. Despite the CHC program being well accessed, increasing systematic differences in health and the social gradient of health inequities are also observed in this country [21]. In response, a postnatal extended home visiting program for new parents that incorporates the principles of proportionate universalism and nurturing care was initiated in the socioeconomically disadvantaged area of Rinkeby in Stockholm, Sweden in 2013. Home visiting programs seek to improve parents’ knowledge and skills and also target contextual factors affecting families living in disadvantages, such as economic independence, social inclusion, and networking [22]. Previous research has shown short- and long-term positive effects on conditions important for children’s early development [23–25]. There is evidence for the effectiveness of home visiting in infancy and early childhood to families in socio-economically disadvantaged areas, and an expanded number of visits can lead to better outcomes [25].

The intervention developed in Rinkeby was composed of additional home visits offered to all first-time parents within the CHC program. The visits were delivered by the same CHC nurse that was also attending the family in the standard CHC program, which at the time included one home visit, but was extended to include five extra home visits. The six home visits were delivered in collaboration with the municipal preventive social services, by teams comprising one CHC nurse and one social worker. Together the teams aimed to offer qualified support to strengthen parenting skills, and to promote child health and development as well as parent health and wellbeing. The content was gradually developed by the professionals during the implementation of the first three-year pilot cycle. Subsequent evaluation established five core components in the Rinkeby program theory: Extra time in the family’s home setting, Qualified team in practical collaboration, Flexible contents in a comprehensive ECD frame, Focus on the child and caretakers from a strengthening perspective, and Multifaceted health promotion and prevention [26]. Details of the program development and content have been described elsewhere [26, 27]. The program was well-received by families with over 90% enrolment and a total of 84% of planned visits carried out during the pilot cycle 2013–2016 [28].

In 2018 the Rinkeby model was taken to scale in the city of Gothenburg through a decision by the healthcare sector and municipal social services in the region of Västra Götaland. The program was implemented in seven CHC centers situated in socioeconomically disadvantaged areas of the city. Gothenburg is the second largest city in Sweden. Similar to other metropolitan areas, inequalities in health and living conditions exist between the populations in different geographic areas of the city, and socioeconomic and ethnic segregation coincide to a large degree [29].Average life expectancy at birth differs by 9 years for women and 8.7 years for men between geographic areas. The proportion of households with children under the age of 19 living under financial strain is on average 22,5% and range from 1% to 55% [29]. Reaching families with the national CHC program is more challenging in the socioeconomically disadvantaged areas. For example, participation rates are lower for follow-up meetings related to post-natal depression and fewer post-natal home visits are carried out, and children present poorer health indicators, such as exposure to smoking in the home, higher levels of overweight and obesity, and lower rates of vaccination coverage [30]. Indicators of inequalities in educational attainment are also noted, where 93% of children of parents with a high level of education qualify for vocational upper secondary school programs, whilst only 49% of children of parents with a low level of education qualify [29].

Considering the above-mentioned challenges in scaling-up ECD interventions and translating proportionate universalism into practice, the present study aimed to investigate the delivery of home visits during the scale-up of the Rinkeby home visiting model in socio-economically disadvantaged areas in the city of Gothenburg, Sweden. Specifically, the study sought to answer: How do professionals perceive their ability to implement the core components of the model? What relevant aspects regarding health equity and the principles of proportionate universalism may be observed?

## Methods

### Study context and participants

The implementation of the extended home visiting program in Gothenburg included seven CHC centers and the three social services branches collaborating with the CHC centers during the study period of December 2018 and June 2022. Three of the CHC centers participated during part of the period, with one center leaving the program in 2020 due to staff shortages and two centers joining the same year. All participating areas were classified as “vulnerable” or “especially vulnerable” in the classification system developed by the Swedish law enforcement authorities. These areas are characterized by socioeconomic hardship, disenfranchisement, lack of trust in social systems, higher levels of crime and risk for erosion of social order [31]. The program in Gothenburg was not only offered to all first-time parents, as in Rinkeby, but also to all families with at least one parent having their first child in Sweden, even if they already had children who were born outside of Sweden. No additional selection criteria, such as medical condition or family need, were used for eligibility for participation. In 2018, two home visits were part of the regular CHC services in the region of Västra Götaland and the extended home visiting program thus implied adding four home visits. During the study period approximately 65 professionals delivered home visits to around 600 families participating in the program.

### Study design

**Fig. 1:**
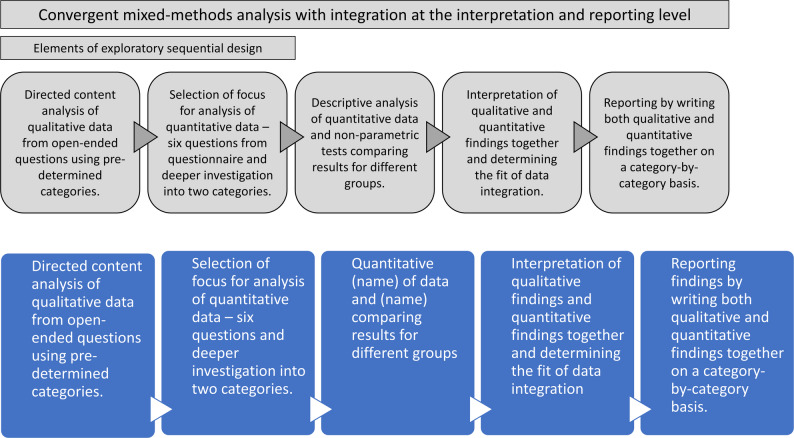
Illustration of the mixed-methods analysis process applied in the study (developed by the authors)

The study was carried out using a convergent mixed-methods approach where the aim was to merge and compare the qualitative and quantitative findings [32]. Elements of an exploratory sequential design were also applied as the qualitative data were analyzed in a first step and the findings then guided selection and analysis of the quantitative data (Fig. 1) [32]. The study was reported according to the Good Reporting of a Mixed Methods study [33]; a completed checklist is available in Supplemental file 1.

### Data collection

All data were collected from nurses and social workers using a questionnaire called “the home visiting Snapshot form”, a modified version of that used by Schachner et al. [34] (Supplementary material 2 in Swedish and Supplementary material 3 in English). The form was inspired by previous research looking at replication of home visiting programs with fidelity by monitoring structural and dynamic characteristics of home visits [35–37]. The Snapshot form was adapted to reflect cultural and program characteristics using a collaborative process between those overseeing the implementation, stakeholders, and representatives for professionals who would be conducting home visits in Gothenburg. Professional teams from one CHC location piloted the form during a two-month start-up period December 2018 - January 2019, after which only minor adjustments were made. The Snapshot form for this study contains questions for the professionals to estimate the amount of content delivered, degree of satisfaction with their own ability to deliver the program strategies, referrals made to community services and/or more intensive healthcare of family support services, the quality of the alliance with parents, and the emotional climate during the visit. It also included two open-ended questions to capture general perceptions of facilitators and barriers in working with the program. The Snapshot form did not collect identifying information about families or professionals other than professional role as a nurse or social worker and CHC location, which limits the possibilities of estimating the total number of families or professionals that participated in this study. Professionals were encouraged to complete the form after every home visit between December 2018 and June 2022. All data were gathered digital using the Esmaker software (Entergate AB, Halmstad, Sweden).

### Qualitative analysis

The qualitative analysis was carried out on the answers to the two open-ended questions, Q1 Overall, what worked well during the visit? and Q2 What do you consider to be needs or areas to develop further? A directed content analysis [38] was used. Coding was performed by MB. Approximately 10% of the answers to question Q1 were coded in the first step and sorted into preliminary categories based on the core components of the Rinkeby home visiting model [20]. The categories were adjusted according to the findings of the first step. Approximately 10% of the responses to question Q2 were then coded using existing codes and adding new ones where needed, and then tentatively sorted into the same categories. After review by ÅN and JL, the categories were considered suitable for both questions and all remaining data were coded and sorted accordingly. Additional codes were added when necessary and sorted into the categories at the end of the coding process. A new category named Home visiting during Covid-19 was added and some adjustments of codes and categories were made in a last step of the analysis.

### Quantitative analysis

Based on the findings of the qualitative analysis, six questions were selected from the Snapshot form (Supplementary material 2) to be included in the present study (Table 1). The response alternatives for three of the questions consisted of Likert rating scales. For those questions the responses were considered as ordinal or categorical variables. Further analyses, including Chi-Square test of independence and Mann-Whitney U-test, were used to determine if the findings were statistically significant. Two of the questions, regarding perception of alliance and emotional climate, were assessed through more than one rating scale in the Snapshot form. Unweighted indexes were created for each question and Mann-Whitney U tests were applied for more comprehensive interpretation of the themes.

**Table 1:** Questions included in the quantitative analysis

Question	Alternatives
Program content that was discussed during the home visit	relationship and interaction with the child; breastfeeding and food; child development and stimulation; relationship and collaboration between parents; child safety; child infections and self-care; routines for feeding and sleep. (Response alternatives for each theme were: less than 25%, about 50%, more than 50% of the visit. For the purposes of the analysis, responses were categorized as delivered or not, regardless of amount of time devoted)
How satisfied are you with your ability to…	focus on the parents’ experience of the child and parenthood; give priority to the parents’ own questions; give attention to the interplay between parents and child; highlight mother’s importance; highlight father’s/other parent’s importance; highlight parent strengths and competencies; be open to the family’s situation (culture and context); identify needs for extra support? (Each alternative rated on a scale from 1–7 anchored by 1 = not at all satisfied and 7 = very satisfied.
How satisfied are you with your ability to develop good collaboration with your colleague?	Rated on a scale from 1–7 anchored by 1 = not at all satisfied and 7 = very satisfied.
How satisfied are you with your ability to apply the home visiting model?	Rated on a scale from 1–7 anchored by 1 = not at all satisfied and 7 = very satisfied.
How do you perceive your alliance with the parent(s)?	Three scales (1–10): Tense-Relaxed; Distanced-Warm; Hard to collaborate-Easy to collaborate. (Responses were indexed without weights for the analysis)
How would you rate the emotional climate during the home visit?	Two scales (1–10): Anxious/unfocused-Calm/focused; Disengaged/little interest-Engaged/great interest. (Responses were indexed without weights for the analysis)

The qualitative analysis also indicated two areas where further quantitative analyses might be relevant: Communication during the visits, and The family’s participation. Subsequently analyses of differences in content delivered during visits with and visits without an interpreter, and visits with one versus two parents present, were conducted using Pearson’s chi squared test. Analyses of differences in professionals’ degree of satisfaction with their own abilities during visits with and without an interpreter, and during visits with one versus two parents present, were conducted using Mann-Whitney U-test. Finally, the Mann-Whitney U-test was also applied to analyze differences in perceptions of alliance with parents and emotional climate during visit during visits with and without interpreter, and for visits with one versus two parents present. For all analyses, the alpha level was set at 0.95.

### Integration of qualitative and quantitative data

The quantitative and qualitative findings were integrated at the interpretation level through a final analysis carried out by MB and MW. The integrated findings are reported through a narrative that uses a weaving approach to report the qualitative and quantitative findings together, following the structure of the qualitative categories [32].

## Results

A total of 3028 Snapshot forms were retrieved, which was the total amount of completed forms during the study period. Extrapolation based on previously published data on number of home visits delivered during the Gothenburg implementation [39] reflects the rate of submission of Snapshot forms to be at approximately 67% of the total number of visits delivered. The two open-ended questions, used for the qualitative analysis, received 1055 and 843 answers, respectively.

The characteristics of the Snapshot forms included in the study are presented in Table 2.

**Table 2:** Characteristics of included snapshot forms

	*N*	Percent
Total no. of Snapshot forms	3028	100%
Completed by CHC nurse	1570	52%
Completed by social worker	1454	48%
Forms from visits with a nurse present	2982	98%
Forms from visits with a social worker present	2749	91%
Visit No. 1	651	21%
Visit No. 2	684	23%
Visit No. 3	577	19%
Visit No. 4	428	14%
Visit No. 5	362	12%
Visit No. 6	326	11%
Visits in the home	1684	56%
Visits at CHC center	1344	44%
Duration 60–75 min	2743	91%
Duration > 75 min	285	9%
Both caregivers present	1612	53%
One caregiver present	1378	46%
Missing data on caregivers present	38	1%
Fathers present	1672	55%
Interpreter present	894	30%
Interpreter not present	2134	70%
Single parent	225	7%
Not single parent	2766	91%
Missing data on single parent	37	1%

The Snapshot form did not collect identifying information about the families or professionals. However, demographic characteristics have been reported in other parallel studies of the Gothenburg home visiting program [40]. In a survey study with 317 parents from 192 families participating in the home visiting program, the mean age was 31 years, 29% were born in Sweden and the sample contained parents from 55 different countries. Of those born abroad 75% had moved to Sweden as adults and 51% had been living in the country five years or less. Level of education varied from 0 to more than 12 years, 57% having studied for over 12 years [40].

The qualitative analysis rendered eight categories that are presented in relation to the core components in the Rinkeby model program theory in Table 3. To illustrate each category, quotes from Q1 and Q2 in the qualitative dataset are provided in text. Quotes are referred to with the following reference (Category, Quote – Question in dataset).

**Table 3:** Analysis categories in relation to the core components of the Rinkeby home visiting program theory

Categories of the analysis	Core components of the Rinkeby model
Visits in the home	Extra time in the family’s home situation
Working with a family focus	Focus on child and caregiver from a strengthening perspective Multifaceted health promotion and prevention
Collaborating as a team	Qualified CHC nurse and social worker in practical collaboration
Applying the home visiting model	Flexible content in comprehensive ECD framework
The family’s participation and engagement	High degree of parent participation in all home visits
Communication during the visits	
Building a trustful alliance	A trustful alliance between parents and professionals
Home visiting during Covid-19	(Not a core component in the Rinkeby model)

### Visits in the home

Compared to meetings at the CHC center or at the social service office, the home visits were described as providing a calm environment where the parents were perceived to be more relaxed. The visits could be carried out at a slower pace which contributed to an open conversation between parents and professionals. Meeting the child in their home environment was also seen as a positive aspect. “The child is calm and secure, easy to observe how they move, play, and respond to new persons (C1Q2-Q1)”. For many visits, it was noted that there was enough time to listen and answer parents’ questions without stress, but there were also descriptions of an experience of time constraints, especially when an interpreter was needed.

### Home visiting during Covid-19

The Covid-19 pandemic during the study period had a considerable negative impact on the possibility to deliver the program in the home environment. While before Covid-19, 90% of the meetings took place in the home, during the pandemic it amounted to 42% of the visits. The pandemic also presented some barriers to professionals’ ability to deliver certain aspects of the program with fidelity. Staff absenteeism due to Covid-19 was perceived as having a negative impact on the work with the program. Still, the professionals to a large degree managed to find alternatives to keep the program running. “Considering the circumstances, meeting at the family center with phone interpretation and protective gear, it worked well and a good atmosphere” (C2Q3-Q1). Another barrier the professionals experienced due to Covid-19 was difficulties in referring families to other services and activities, as most were closed. The pandemic-related closures in the community were perceived to increase families’ social isolation and worry, and it negatively affected the program goal of helping families integrate into the community. “The mother has no relatives or friends yet in Sweden apart from her husband’s family. The pandemic, amongst other things, has made it impossible for her to start Swedish classes” (C2Q5-Q2).

### Collaborating as a team

The program’s aim to offer home visits by two skilled professionals was achieved to a large degree. A CHC nurse was present in 98% of the visits and a social worker was present in 91% of the visits. Teamwork in itself was also one of the most pronounced positive achievements, especially in relation to the core program strategy of creating a joint conversation with the family and one in which both professionals contribute their perspectives and competencies. “We listened and opened up for each other in the conversation. We followed the parents in their questions and picked up the different issues they talked about” (C3Q1-Q1). Attention to parents’ needs, wishes and concerns was commonly mentioned as something that worked well in the team. The professionals also noted their needs for skills-development to improve the balance between stimulating parents’ active participation and delivering information. “To get them to describe more how they do things with the child. I find it hard to get a conversation going around the different issues. It is easy to feel that we give a lot of information” (C3Q3-Q2). In the Snapshot form the professionals estimated the level of satisfaction with their ability to create a good collaboration with their colleague during each home visit. The average score was very high, reaching an average of 6.1 on a scale of 1–7.

### Applying the home visiting model

The professionals’ ratings of the level of satisfaction related to their own ability to apply the home visiting model were also high, gradually increasing from an average of 5.8 at the first home visit, to 6.2 at the last visit, on a scale from 1 to 7. They reported that a positive aspect was how the model gives room for flexibility within the framework of the six visits. They also appreciated the pedagogical aids such as a content guide, pictures and dolls. Still, the situation-based method required new competencies. “How does one handle unplanned situations, such as a friend turning up in the middle of the home visit?” (C4Q4-Q2). Despite the perceived flexibility of the model, there were some experiences of too much content to be covered in a short time during the visits. The professionals also highlighted the need to adapt the model to become relevant for the target group of mothers who already have children but are having their first child in Sweden. “It can be hard to engage this mother who already has a child and is very competent. We feel that she doesn’t think we bring anything new” (C4Q6-Q2).

### Working with a family focus

The professionals’ focus on the family was evident in several ways. There were many observations of families’ situations, positive developments, concerns and needs for change. “Good conversation and collaboration between parents. The father is shyer with a lot of integrity and the mother is very open. They seem to be well, both in relation to the child, psychologically and in the adult relationship (C5Q1-Q1)”. “Talk about difficult moments with the child and strategies to handle them, as well as collaboration between the parents” (C5Q2-Q1). Professionals also frequently described how the conversation centered around the child during the visit, along with parenting challenges and areas to work on with the parents. The professionals reflected on their interventions during the visits and how they wanted to improve. “I can get better at emphasizing how important the parents are for the child, more clearly than I do now (C5Q4-Q2)”. Table 4 illustrates that the family focus was applied through a range of themes addressed during the home visits, including relational and practical.

**Table 4:** Themes that were discussed during the total of home visits

Theme	Visits with theme discussed (percent)
Relationship and interaction with the child	80%
Breastfeeding and food	77%
Child development and stimulation	74%
Couple relationship and collaboration	64%
Routines for feeding and sleep	64%
Child safety	48%
Child infections and selfcare	41%

### Communication during the visits

The importance of and challenges involved in communication with parents was an issue raised by the professionals, and it mainly concerned language barriers. Many parents spoke little, or no Swedish and the lack of a common language was described to interfere in the visits. The most used strategy was to hire an interpreter, either to attend the visit in person or to translate over the phone. A total of 30% of the visits were carried out with an interpreter. In several cases there was praise for the professionalism of the interpreters, but more often, there was frustration among the home visiting staff, especially regarding phone translation. It was perceived that the translating limited the possibility to follow the work method and fully deliver the home visiting model. “It is very hard to work with reflective questions with an interpreter. It is easier when the issues and questions are more informative. The quality can just not be the same through an interpreter” (C6Q3-Q2). Another solution was for one parent to translate for the other one, but the professionals observed that this could limit both parents from having their voices heard in the conversation. Sometimes the absence of an interpreter pushed the parents to dare to try their Swedish and ended up in a positive experience. On other occasions, parents seemed uncomfortable and declined the offer of an interpreter, which resulted in situations where limitations in the communication could cause misunderstandings between parents and professionals.

The analysis of the number of themes discussed, comparing home visits with and without interpreter, shows that significantly less content was delivered during the home visits with an interpreter present (Table 5).

**Table 5:** Comparison of content delivered during visits with versus without interpreters

Theme	Visits with interpreter (total of 894 questionnaires) and theme discussed *N* (%)	Visits without interpreter (total of 2134 questionnaires) and theme discussed *N* (%)	Pearson Chi-Square	*P*-value at 95% confidence level
Relationship and interaction with the child	661 (74)	1755 (82)	26.9	< 0.001
Breastfeeding and food	681 (76)	1662 (78)	1.0	0.306
Child development and stimulation	613 (69)	1620 (76)	17.6	< 0.001
Routines for feeding and sleep	495 (55)	1440 (67)	40.0	< 0.001
Couple relationship and collaboration	484 (54)	1464 (69)	57.5	< 0.001
Child safety	371 (42)	1079 (51)	20.7	< 0.001
Child infections and selfcare	314 (35)	920 (53)	16.7	< 0.001

A similar result was found regarding the question of how satisfied the professionals were with their own ability to apply the strategies of the program. The ratings are significantly lower for visits with interpreters (Table 6).

**Table 6:** Comparison of professionals’ satisfaction with abilities during visits with versus without interpreters

Professionals’ level of satisfaction with own ability to… (score 1–7)	Visits with interpreter, mean and (median)	Visits without interpreter, mean and (median)	Mann-Whitney U test Z-value	*P*-value at 95% confidence level
… focus on the parents’ experience of the child and parenthood	5.5 (6)	6.1 (6)	-13.3	< 0.001
…prioritize the parents’ own questions	6.0 (6)	34 (6)	-9.8	< 0.001
…give attention to the parent-child interaction	5.6 (6)	6.1 (6)	-11.2	< 0.001
…highlight mother’s importance	5.4 (6)	5.8 (6)	-8.8	< 0.001
… highlight father’s/other parent’s importance	5.0 (5)	5.6 (6)	-10.4	< 0.001
… highlight parents’ strengths and competencies	5.5 (6)	5.9 (6)	-9.3	< 0.001
…be open to the family’s situation (culture and context)	5.8 (6)	6.2 (6)	-10.7	< 0.001
… identify needs for extra support	5.6 (6)	6.1 (6)	-12.1	< 0.001

### The family’s participation and engagement

The professionals documented whether both parents were present during the home visit, how parents engaged with the content and the extent to which parents contributed to the joint conversation. Meeting with two participating parents perceived as active, open, and interested with many questions was much appreciated by the professionals. “When both parents are there, there is another dynamic in the dialogue (C7Q1-Q1)”. “The parents are very engaged and proud, want to show us the child’s development. Made it easier for us to talk” (C7Q2-Q1). When parents were quieter it was taken as a challenge by the professionals. “To make the parents express themselves more, they often answered that all was well and had difficulty in telling more (C7Q3-Q2)”. It was also noted that some parents had difficulties in reflecting on their own parenting and that the professionals felt they needed to act on this. “Capacity to reflect, how do you teach this to someone? So hard with this parent who may not understand why she should receive home visits […]. To get questions that they do not know what answer we want. That they do not have any questions themselves. Dilemma (C7Q4-Q2)”.

Both parents were present in 72% of the first home visit, while in the remaining five visits, two parents participated in approximately half of the visits (Table 7).

**Table 7:** Percentage of home visits with both parents present

	Both parents present (percent)
Home visit 1	72%
Home visit 2	54%
Home visit 3	50%
Home visit 4	46%
Home visit 5	43%
Home visit 6	42%

According to professionals’ documentation, the continued engagement of both parents could be perceived as a barrier in need of solutions.

“How can we engage the parents who do not participate in the visits anymore? We talked about finding a time that suits the other parent, but it has been hard due to their irregular work hours (C7Q5-Q2)”. The absence of fathers was frequently observed as a problem. The professionals strove to promote fathers’ attendance, to give them attention and to motivate them when they were present. “As it was his first visit, we put extra effort in involving him in the conversation and feel important (C7Q6-Q1)”.

The analysis of the number of themes discussed, comparing home visits with one parent versus both parents present, shows that significantly less content was delivered during the home visits with only one parent present (Table 8).

**Table 8:** Comparison of content delivered during visits with one versus two parents present

Theme	Visits with one parent (total of 1378 questionnaires) and theme discussed *N* (%)	Visits with two parents (total of 1612 questionnaires) and theme discussed *N* (%)	Pearson Chi-Square	*P*-value at 95% confidence level
Relationship and interaction with the child	1053 (76)	1330 (83)	17.0	< 0.001
Breastfeeding and food	1010 (73)	1302 (81)	23.7	< 0.001
Child development and stimulation	1006 (73)	1201 (75)	0.9	0.353
Routines for feeding and sleep	845 (61)	1065 (66)	7.3	0.007
Couple relationship and collaboration	734 (53)	1188 (74)	135.1	< 0.001
Child safety	591 (43)	840 (52)	25.3	< 0.001
Child infections and selfcare	470 (34)	751 (47)	47.9	< 0.001

Similar results were found regarding the professionals’ satisfaction with their ability to apply the strategies in the model. All ratings except for one variable are significantly lower for visits with one parent present (Table 9).

**Table 9:** Comparison of professionals’ satisfaction with their abilities during visits with one or two parents present

Professionals’ level of satisfaction with own ability to… (score 1–7)	Visits with one parent, mean and (median)	Visits with two parents, mean and (median)	Mann-Whitney U test Z-value	*P*-value at 95% confidence level
…focus on the parents’ experience of the child and parenthood	5.8 (6)	6.0 (6)	-5.8	< 0.001
… prioritize parents’ own questions	6.1 (6)	6.4 (7)	-6.7	< 0.001
…give attention to the parent-child interaction	5.8 (6)	6.0 (6)	-4.7	< 0.001
…highlight mother’s importance	5.7 (6)	5.7 (6)	-0.0	0.962
… highlight father’s/other parent’s importance	5.0 (5)	5.8 (6)	-16.9	< 0.001
… highlight parents’ strengths and competencies	5.6 (6)	5.8 (6)	-4.9	< 0.001
…be open to the family’s situation (culture and context)	6.0 (6)	6.2 (6)	-5.8	< 0.001
… identify needs for extra support	5.8 (6)	6.1 (6)	-5.8	< 0.001

### Building a trustful alliance

The professionals appeared generally satisfied with the alliance they established with parents and the quality of the alliance was often associated with parents’ active participation and the quality of the conversations during the home visits. “The family had many questions that we could answer together. We have built a good relationship and trust. Good, the father is very open and shares things and is receptive to information. The mother is shyer but is receptive to information. They both seek information and are engaged. The family felt secure with us coming there […] (C8Q1-Q1)”. The perception of trustful alliance was also connected to the recurring contact over time which created the possibility of getting to know each other. Parents’ sharing of sensitive issues also seemed to be an indicator of trust and satisfaction with the program. “A good conversation where both parents participate on equal terms. Very positive relationship with the family. They appreciate the home visits (C8Q4-Q1)”. Only a few observations of difficulties in building trust were made. These were related to perceived problems with establishing contact, communication, and collaboration with the parents. The quantitative data confirms the general positive view on building trustful alliances with parents. The five dimensions of quality of the relationship and emotional climate were rated with averages between 8.12 and 8.80 (on a scale from 1 to 10) for all visits.

However, differences in ratings of the alliance and emotional climate during visits with and without interpreters were once again evident. Ratings of alliance and emotional climate during visits were significantly lower when interpreters were present (Table 10).

**Table 10:** Comparison of alliance and emotional climate during visits with and without interpreter

Dimension (scale 1–10)	Visits with interpreter, mean and (median)	Visits without interpreter, mean and (median)	Mann-Whitney U test Z-value	*P*-value at 95% confidence level
Professionals’ perception of the alliance with the parent(s) during the visit	7.7 (8.0)	8.4 (9.0)	-11.3	< 0.001
Professionals’ rating of the emotional climate during the visit	8.0 (8.0)	8.7 (9.0)	-12.8	< 0.001

Similarly, significantly lower ratings of alliance and emotional climate were given for visits with one parent present than for those with both parents present (Table 11).

**Table 11:** Comparison of relation and emotional climate during visits with one versus two parents present

Dimension (scale 1–10)	Visits with one parent, mean and (median)	Visits with two parents, mean and (median)	Mann-Whitney U test Z-value	*P*-value at 95% confidence level
Professionals’ perception of the alliance with the parent(s) during the visit	8.0 (8.0)	8.3 (8.7)	-6.2	< 0.001
Professionals’ rating of the emotional climate during the visit	8.3 (8.5)	8.6 (9.0)	-7.9	< 0.001

## Discussion

### Applying the program’s core components

The qualitative content analysis of the Gothenburg professionals’ registration of home visits was, in its first step, directed by the core components of the original Rinkeby program model [26]. The data indicated that the same or very similar components also characterized the central categories of the Gothenburg documentation, suggesting that adequate fidelity to the original program was largely achieved. The professionals considered their ability to apply the Rinkeby model to be very successful in several areas, including working within the program’s framework, working in professional teams and establishing a trustful alliance with the families. With regard to the delivery of content, all five components of the nurturing care framework [4] were represented to a large degree.

Some specific challenges in applying the model were also noted by the professionals, for example the need to adapt content for mothers who already had a previous child, as well as the challenges of applying the interactive work method with parents who were shy, quiet or presented difficulties in reflective practices.

Two additional categories that were not present in the original program core components were developed through the analysis: one was related to the program implementation during the Covid-19 pandemic, and the other captured the importance and challenges of communication when delivering home visits. The issues identified in relation to communication are discussed further below along with findings with a possible bearing on health equity and the principle of proportionate universalism.

### Communication affects content delivered and alliance

The importance of establishing a good communication to successfully deliver the program was one aspect that became apparent in the analysis. Language barriers were common and the predominant solution of employing an interpreter, in 30% of the visits, was oftentimes viewed as a challenge. Compared to home visits delivered without a translator, visits delivered with a translator were not only associated with less content being delivered, but also lower ratings of the alliance and emotional climate, as well as professionals’ experience of a more limited ability to apply the strategies of the program. These findings indicate the risk for inequitable services; therefore, a thoughtful exploration of possible solutions and barriers associated with the need for translation is of utmost importance.

The challenges and risks that language barriers introduce in health care delivery warrant discussion. Swedish law mandates that translation services must be provided to patients when there is need [41, 42]. Our results suggest that parents and professionals could have differing perceptions about the need for interpreters, as seen in cases where parents declined this service when the professionals had deemed it necessary. Working with authorized interpreters also seems to be considered the most viable solution employed by healthcare staff to overcome communication barriers [43], but it entails a number of challenges. The physical presence of an interpreter may interfere during home visits in crowded housing situations with many family members present [44]. Substituting an in-person interpreter with one via telephone, while a solution in some ways, may decrease the service quality and increase the risks of misunderstandings [45]. Interpreters are not always available when needed [44, 46, 47] and authorized interpretation does not automatically guarantee skill in medical interpreting [43, 48]. Using professional interpreter services can have a negative impact on establishing trust and parents’ willingness to disclose private issues [43, 45–47] Parents who are not comfortable having an interpreter present might prefer using a relative to translate [49, 50], but this solution could also bring the risk of a poor quality translation and might hinder the disclosure of intimate issues [46, 47]. For natural reasons, visits involving translation require more time, which may result in less program content delivery, as was found in the present study. Studies have also reported how lack of continuity of interpreters in programs have had negative effects on the service quality [46, 47]. An evaluation report of the Rinkeby home visiting program described initial efforts to train specific interpreters for the intervention but that this was rendered unfeasible due to high turnover [51].

While no ideal solution to the barriers of working with translation seems to exist, several alternatives have been discussed in the literature. Working effectively with interpretation requires good planning of content delivery and staff training in interaction techniques [45, 46], something that has been requested by home visiting staff [44]. An alternative would be to use bilingual healthcare professionals during home visits [49, 52]. While having the potential to be a good strategy, hiring bilingual staff may not be feasible in diverse contexts such as Gothenburg with parents from over 50 nationalities. Another possibility is the use of cultural brokers in the form of doulas, a solution recommended for aiding both language translation and sensitivity to cultural issues that may be underlying communication challenges [47, 49, 53]. The use of language translation apps to facilitate communication between parents and professionals seems to be common, and although not considered a substitute for professional translation it is recognized as encouraging parents to be more active in the interaction [50]. Access to targeted multimedia resources such as pictures and videos is also a way of supporting direct communication between parents and professionals [50]. Staff training in working with interpreters and the use of technology need to be studied further in relation to reduced disparities in content delivery when implementing the program model.

### The professionals strive to engage fathers

The importance of fathers’ participation during the home visits is one of the primary goals of the home visiting program, and this was embraced by the professionals in Gothenburg. They strove to motivate fathers’ participation during the visits and showed positive encouragement when they attended. Our results thus stand in contrast to other studies showing that male caregivers are frequently neither targeted nor actively included in ECD interventions [54–56]. Disparities in attention to fathers’ relative to mothers’ involvement persist despite clear evidence indicating that engaging fathers has a positive influence on child development [56, 57]. Positive outcomes associated with fathers’ involvement include strengthening of fathers’ own parenting [54, 56, 58], improvement of fathers’ gender attitudes [59], benefits for the whole family and increased family retention in ECD programs [56, 60, 61]. However, similar to findings for other parenting interventions, professionals working with the home visiting program in Gothenburg reported experiencing difficulty retaining fathers’ during the program duration. The professionals in our study generally related fathers’ absence with work engagement, which is in line with other studies [54, 61–64]. An earlier study from the Rinkeby home visiting program found that this especially affected those fathers experiencing irregular or informal work situations, who did not have the power to demand time off work to participate in the home visits [65]. Flexible home visiting schedules are thus central to enable the participation of all fathers, and professionals need support within their agencies to tailor their availability so that the needs of all families are accommodated.

Cultural barriers to father involvement have been discussed in the literature but they seem less present in the Gothenburg professionals’ reflections. Research highlights how fathers may not participate due to perceptions of roles and gender biases held by themselves, the child’s mother as well as professionals, or contained in program structure and content [61, 62]. The families reached by the Gothenburg home visiting program represent many different cultures and the suggestion to strive within the program for a deeper awareness of existing norms and fatherhood roles in the community [61] could be relevant. It has been noted that parenting interventions in early life represent an opportunity to involve fathers and address gender issues, but that it is rarely explored [59]. Additional facilitating factors for father involvement include collaboration with stakeholders in the community, the use of media to promote program and recruit fathers, and to work with peer-engagement among fathers and participatory methods, for example involving fathers in designing the program content [54].

### Leaving no one behind

The present study has revealed overall high ratings by the home visiting professionals on several indicators of program fidelity such as delivery of program content, satisfaction with their abilities and teamwork, as well as the establishment of a good alliance with parents and a favorable climate during home visits. This can lead us to draw positive conclusions regarding successful implementation of the Rinkeby model in Gothenburg. Parallel studies of the Gothenburg home visiting program based on surveys and interviews with parents reveal equally positive findings in terms of parents’ satisfaction with the program, its content and the professionals [40]. Our analysis, however, also reveals systematic differences when we compare home visits with different characteristics. During home visits with interpreters present the professionals estimated that less content was delivered. They were less satisfied with their own abilities and gave lower ratings for the alliance with parents and emotional climate of the visits. The same pattern was observed during those visits with only one parent present compared to visits with both parents. These rather small but statistically significant differences are relevant to consider through the lens of health equity. The Marmot report [7] states that without a specific focus on equity, universal health interventions tend to result in greater uptake by groups with stronger resources, leaving the more vulnerable groups behind. A recent study on the inverse care law [66] also indicates that a disproportionate care law persists in high-income countries whereby groups of social disadvantage do receive a larger amount of health care, but of worse quality and still not in sufficient quantity to meet their needs [67]. Through our findings we could only speculate in whether this kind of forces are also at play in the implementation of the home visiting program in Gothenburg. Still, within an intervention built upon proportionate universalism it is crucial to pay attention to such systematic differences that could indicate that some groups that possibly have higher needs are actually getting less. Further research is warranted here.

It has been pointed out that in countries with high standard health-care safety nets, more consideration should be given to co-design and adaptation to context in order to improve home visiting interventions [68]. Focus on support to professionals and implementation are also regarded as central [68]. While co-creation involving the professionals in Rinkeby was an important aspect of developing the original home visiting model [27], the professionals did not have the same opportunity when the model was adopted to the Gothenburg context. Engaging staff as well as the target population in co-creation is a potential that could still be explored in the future development of the home visiting program in Gothenburg. Continuous monitoring of implementation indicators is also essential [12, 14] to ensuring that program content and quality is equitable for all participants.

### Strengths and weaknesses

This study has several strengths. Firstly, the mixed-methods approach gave the study the depth and breadth necessary for exploring subtle barriers to implementing the extended home visiting program. The qualitative dimension of the study amplifies the professionals’ perspective. The quantitative dimension enhanced the professionals’ perspective, and further strengthened that their impressions of differences were in fact statistically significant.

The use of the Snapshot form enabled the collection of representative amounts of quantitative and qualitative data which were provided voluntarily by the professionals. During the preparation phase of implementation, key stakeholders expressed concerns that the fidelity monitoring, although important, might be too time consuming and burdensome to carry out [69]. This proved not to be the case.

The study also had some apparent weaknesses. The unpredictable event of the Covid-19 pandemic affected the possibility to deliver the program as intended, for instance the number of visits in the home versus at the CHC clinic. This might have influenced the data and subsequent findings. Another limitation is the lack of information about our participants as a consequence of maintaining the Snapshot responses as anonymous and voluntary. We do not know the exact number of professionals and families included in the study, nor do we know the demographic composition of the groups. We do not know whether multiple forms submitted from visits with the same families could have an impact on the results. Another possible limitation is that we did not study differences between professional groups’ (nurses and social workers) ratings or comments on the Snapshot form, nor did we analyze in depth the potential influence of time on professionals’ ratings. Therefore, it is not known whether there were substantial changes in professionals’ experience with the program over time that may have affected ratings, and whether such trends would have influenced the results. We suggest that investigation of additional perspectives on responses to the Snapshot form could reveal further knowledge about program implementation and pinpoint additional areas for improvement.

## Conclusions

This study contributes to getting one step closer to more equitable conditions for children’s health and well-being by highlighting the critical importance of monitoring the implementation of evidence based ECD programs aimed at reducing disparities. Overall, professionals’ responses reflect adequate fidelity in terms of perceptions of self-efficacy, establishment of a parent-provider alliance, and content delivered. At the same time, nuanced differences in program delivery based on specific visit characteristics were discovered. Even if the differences in numbers are small, they are significant. This does not mean that the home visits in the lower rated groups are deficient, but they are not as good as they could be. Solutions are called for because systematic differences in health care service delivery contributes to an erosion of health equity for disadvantaged groups over time. Our findings suggest three main areas for improvement of the extended home visiting program studied: (1) strategies to overcome language and communication barriers, perhaps with specialized training or use of technology; (2) supporting opportunities for professionals to include both parents to a higher degree in the home visits; and (3) strategies to deliver program content and model when only one parent is present during the visit. Future research examining improvements in equitable service delivery with the applications of different solutions in these areas is recommended. The Snapshot form could be a useful addition to program materials to monitor future implementations of the program. Ongoing fidelity monitoring has facilitated our ability to study health equity and this is a key take-away for other healthcare settings implementing ECD programs.

## Supplementary Information

Below is the link to the electronic supplementary material.

Supplementary Material 1

Supplementary Material 2

Supplementary Material 3

## Data Availability

Data can be made available by the corresponding author upon reasonable request.

## Abbreviations

CHC: child health care
ECD: early childhood development
