# Supplementary material for: Toward better home visits: a mixed-methods study identifying disparities in early childhood program delivery to promote health equity
Source: BMC Health Serv Res. 2026 Feb 2;26:310. doi: 10.1186/s12913-026-14092-2 (PMC12937560; doi:10.1186/s12913-026-14092-2)
Supplement: Supplementary file 1 — Supplementary Material 1 [file 12913_2026_14092_MOESM1_ESM.pdf]

Good Reporting of A Mixed Methods Study (GRAMMS) checklist

Guideline	Section, page
Describe the justification for using a mixed methods approach to the research question	Background, page 7 and Methods, page 8
Describe the design in terms of the purpose, priority and sequence of methods	Methods, illustration page 9
Describe each method in terms of sampling, data collection and analysis	Methods, page 9-12
Describe where integration has occurred, how it has occurred and who has participated in it	Methods, page 12
Describe any limitation of one method associated with the presence of the other method	Discussion, page 30-31
Describe any insights gained from mixing or integrating methods	Discussion, page 25-30

Reference:

O'Cathain A, Murphy E, Nicholl J. The quality of mixed methods studies in health services research. J Health Serv Res Policy. 2008;13: 92-98.
