# Supplementary material for: Toward better home visits: a mixed-methods study identifying disparities in early childhood program delivery to promote health equity
Source: BMC Health Serv Res. 2026 Feb 2;26:310. doi: 10.1186/s12913-026-14092-2 (PMC12937560; doi:10.1186/s12913-026-14092-2)
Supplement: Supplementary file 2 — Supplementary Material 2 [file 12913_2026_14092_MOESM2_ESM.pdf]

Ögonblicksbild
Utökade hembesök Göteborg

Datum för hembesöket: ________________

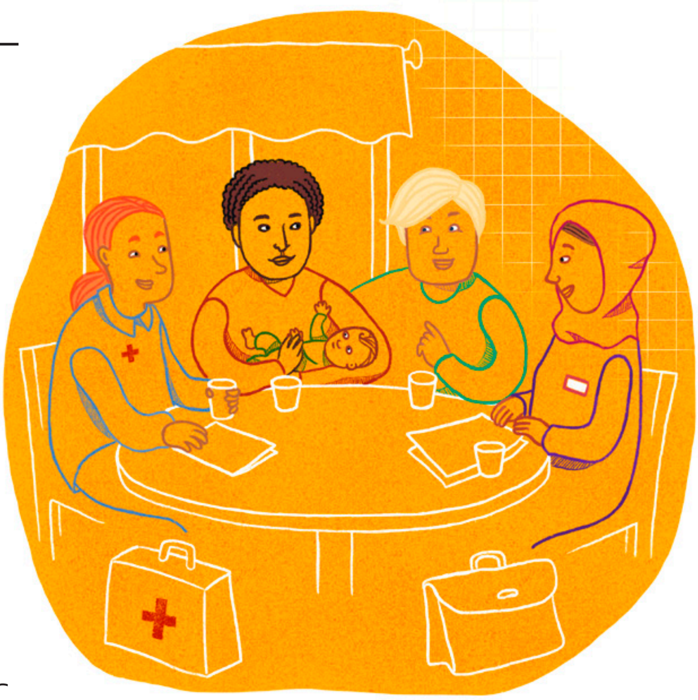

Ditt yrke:
- ☐ BHV-sjuksköterska
- ☐ Socionom

Fyller ni i ögonblicksbilden tillsammans?
- ☐ Ja
- ☐ Nej

BVC:
- ☐ Närhälsan Opaltorget BVC
- ☐ Närhälsan Hjällbo BVC
- ☐ Närhälsan Angered BVC
- ☐ Capio BVC Angered
- ☐ Närhälsan Biskopsgården BVC
- ☐ Nötkärnan Friskväderstorget BVC
- ☐ Närhälsan Lövgärdet BVC/Gårdsten BVC
- ☐ Angereds närsjukhus

Nummer på hembesök:
- ☐ 1
- ☐ 2
- ☐ 3
- ☐ 4
- ☐ 5
- ☐ 6

Besöket äger rum:
- ☐ I hemmet
- ☐ På mottagning

Tidsåtgång på hembesöket:
- ☐ 60–75 minuter
- ☐ >75 minuter

Båda föräldrar deltar under besöket:
☐ Ja ☐ Nej

Föräldern är ensamstående:
☐ Ja ☐ Nej

Ålder på barnet:

☐ 1 v	☐ 2 mån	☐ 6 mån	☐ 10 mån	☐ 14 mån
☐ 2 v	☐ 3 mån	☐ 7 mån	☐ 11 mån	☐ 15 mån
☐ 3 v	☐ 4 mån	☐ 8 mån	☐ 12 mån	☐ 16 mån
☐ 1 mån	☐ 5 mån	☐ 9 mån	☐ 13 mån	☐ >16 mån

Närvarande:
- ☐ ☐ mamma
- ☐ ☐ pappa
- ☐ ☐ annan vårdnadshavare
- ☐ ☐ annan omsorgsgivare
- ☐ ☐ släkting/vän till familjen
- ☐ ☐ ☐ ☐ andra barn

Professioner närvarande vid besöket:
- ☐ BHV-sjuksköterska
- ☐ Socionom
- ☐ Annan profession

Användes tolk?
- ☐ Ja
- ☐ Nej

Samtalsteman som berördes under besöket

	**Kort stund** (<25 % av besöket)	**Ungefär hälften** (ca 50 % av besöket)	**Längre stund** (>50 % av besöket)
Relation och samspel med barnet	☐	☐	☐
Amning och mat	☐	☐	☐
Barnets utveckling och stimulans	☐	☐	☐
Parrelation och samarbete	☐	☐	☐
Barnsäkerhet	☐	☐	☐
Infektioner hos barn och egenvård	☐	☐	☐
Rutiner kring mat och sömn	☐	☐	☐
Annat: ______________________	☐	☐	☐

Hur nöjd är du med din förmåga att ...

	Inte alls nöjd						Mycket nöjd
… lägga tonvikten på föräldrarnas upplevelse av barnet och föräldraskapet?	1	2	3	4	5	6	7
… prioritera föräldrarnas egna frågor?	1	2	3	4	5	6	7
… uppmärksamma samspel mellan föräldrar och barn/föräldrar emellan?	1	2	3	4	5	6	7
… lyfta mammans betydelse för barnet?	1	2	3	4	5	6	7
… lyfta pappans/den andra förälderns betydelse för barnet?	1	2	3	4	5	6	7
… lyfta föräldrarnas kompetenser/styrkor?	1	2	3	4	5	6	7
… vara öppen för familjens egen situation (kultur och kontext)?	1	2	3	4	5	6	7
… identifiera familjens behov av extra stöd?	1	2	3	4	5	6	7
… få till ett samarbete med din kollega/partner?	1	2	3	4	5	6	7
… förhålla dig till Utökade hembesöksmodellen?	1	2	3	4	5	6	7

Föreslog/följde du upp remiss eller hänvisning till någon av följande under besöket:

	Föreslog	Följde upp
Extra kontroll av barnet på BVC/VC/BUM	☐	☐
Kontakt MBHV-psyk/VC/annan sjukvård (förälder)	☐	☐
Folktandvården	☐	☐
Socialtjänst: Barn och familj/spädbarns-verksamhet (service eller bistånd)	☐	☐
Socialtjänst: Försörjningsstöd	☐	☐
Socialtjänst: Boendestöd	☐	☐
Kvinnojour, Kriscentrum för män/kvinnor, Utväg (eller liknande)	☐	☐
Utbildning/ Arbete/ Sysselsättning (förälder)	☐	☐
Migrationsverket/ juridiskt ombud (eller liknande)	☐	☐
Öppen förskola	☐	☐
Kontakt Förskola/(-köassistent mm)	☐	☐
Bibliotek	☐	☐
Annat	☐	☐

	Ja	Nej	Vet ej
SRS (Session Rating Scale) eller liknande har utförts med familjen	☐	☐	☐
Familjen har varit till öppen förskola	☐	☐	☐
Familjen har varit till biblioteket	☐	☐	☐
Familjen har fått besök av Bokstart	☐	☐	☐
Familjen har fått extra samtal med föräldra-rådgivaren	☐	☐	☐
Familjen har tagit del av annan föräldrastöd-sinsats	☐	☐	☐
En orosanmälan har gjorts (av dig eller någon annan)	☐	☐	☐

Hur upplever du din relation med denna förälder/föräldrar?

Spänd	1	2	3	4	5	6	7	8	9	10	Avslappnad
Distanserad	1	2	3	4	5	6	7	8	9	10	Varm
Svårt samarbeta	1	2	3	4	5	6	7	8	9	10	Lätt samarbeta

VÄSTRA GÖTALANDSREGIONEN
Göteborgs Stad

Hur skulle du skatta det känslomässiga klimatet under detta hembesök?

Oroligt/ofokuserat	1	2	3	4	5	6	7	8	9	10	Lugnt/fokuserat
Oengagerat/ litet intresse	1	2	3	4	5	6	7	8	9	10	Engagerat/ stort intresse

Vilka professionella strategier fick du användning för under besöket?

- ☐ Samtal
- ☐ Känslomässigt stöd
- ☐ Rådgivning
- ☐ Praktiskt stöd
- ☐ Krishantering
- ☐ Observation
- ☐ Demonstrera/visa
- ☐ Videoexempel
- ☐ Annat

Reflektionsfrågor:

Överlag, vad fungerade bra under besöket?

Vad ser du för utvecklingsområden/behov?

Något stöd du behöver i handledning utifrån detta besök?

Tack för din medverkan!
