# Supplementary material for: Toward better home visits: a mixed-methods study identifying disparities in early childhood program delivery to promote health equity
Source: BMC Health Serv Res. 2026 Feb 2;26:310. doi: 10.1186/s12913-026-14092-2 (PMC12937560; doi:10.1186/s12913-026-14092-2)
Supplement: Supplementary file 3 — Supplementary Material 3 [file 12913_2026_14092_MOESM3_ESM.pdf]

Supplementary material 3 – English version of Supplementary material 2

Home visiting snapshot form

Date for home visit: ___________

Your profession: □ CHC nurse □ Social worker

CHC location:

Home visit number: □ 1 □ 2 □ 3 □ 4 □ 5 □ 6

Visit location: □ in the home □ at CHC

Length of home visit: □ 60-75 minutes □ >75 minutes

Chilld's age:

□ 1 week	□ 2 month	□ 6 month	□ 10 month	□ 14 month
□ 2 week	□ 3 month	□ 7 month	□ 11 month	□ 15 month
□ 3 week	□ 4 month	□ 8 month	□ 12 month	□ 16 month
□ 1 month	□ 5 month	□ 9 month	□ 13 month	□ > 16 month

Did both parents participate? □ Yes □ No

Is this a single parent: □ Yes □ No

Persons present:

□ □ mother	□ □ other caregiver
□ □ father	□ □ relative/family friend
□ □ other guardian	□ □ □ □ other children

Professionals present during visit:

□ CHC nurse
□ Social worker
□ Other professional

Was an interpreter present? □ Yes □ No

Themes discussed during the home visit:	**Briefly** (<25% of visit)	**About half the visit** (approx. 50% of visit)	**At length** (>50% av visit)
Relationship and interaction with the child	☐	☐	☐
Nursing and food	☐	☐	☐
Child development and stimulation	☐	☐	☐
Couple relationship and cooperation	☐	☐	☐
Child safety	☐	☐	☐
Child infections and self care	☐	☐	☐
Routines for feeding and sleeping	☐	☐	☐
Annat:	☐	☐	☐

How satisfied are you with your ability to …	Not satisfied at all						Extremely satisfied
… focus on parents' experience of the child and parenting?	1	2	3	4	5	6	7
…prioritize the parents' own questions	1	2	3	4	5	6	7

…give attention to the parent-child interaction	1	2	3	4	5	6	7
…highlight mother's importance	1	2	3	4	5	6	7
… highlight father's/other parent's importance	1	2	3	4	5	6	7
… highlight parents' strengths and competencies	1	2	3	4	5	6	7
…be open to the family's situation (culture and context)	1	2	3	4	5	6	7
… identify needs for extra support	1	2	3	4	5	6	7
…prioritize the parents' own questions	1	2	3	4	5	6	7
…work according to the home visiting program	1	2	3	4	5	6	7

Did you refer to/follow-up a referral to or recommend one of the following during the visit:	Recommended	Followed up
Extra health assessment of child at CHC or specialty clinic	☐	☐
Contact with Mother-child health clinic psychologist/Public health clinic/ other healthcare (parent)	☐	☐
Dentistry	☐	☐
Social services: child and family (voluntary or mandatory services)	☐	☐
Social services: Economic support	☐	☐
Social services: Housing	☐	☐
Women's shelter, Crisis center for men/women, or similar	☐	☐
Education/employment (parent)	☐	☐
Department of migration/ legal support (or similar)	☐	☐
Open preschool	☐	☐
Contact preschool/registration assistance	☐	☐
Library	☐	☐

	Don't know	Yes	No
SRS (Session Rating Scale) similar has been used	☐	☐	☐
Family has been to Open preschool	☐	☐	☐
Family has been to the Library	☐	☐	☐
Book Start has visited the family	☐	☐	☐
The family has had extra meetings with the social worker/parent supporter	☐	☐	☐
The family has participated in another parent support program	☐	☐	☐
A worry about the family has been reported to social services (by you or someone else)	☐	☐	☐

How do you perceive your alliance with the parent(s)?											
Tense	1	2	3	4	5	6	7	8	9	10	Relaxed
Distanced	1	2	3	4	5	6	7	8	9	10	Warm
Hard to collaborate	1	2	3	4	5	6	7	8	9	10	Easy to collaborate
**How would you rate the emotional climate during the home visit?**											
Anxious/unfocused	1	2	3	4	5	6	7	8	9	10	Calm/focused
Disengaged/little interest	1	2	3	4	5	6	7	8	9	10	Engaged/great interest

Which professional strategies did you use during the visit?

Discussion □
Emotional support □
Advising □

Practical support □
Crisis intervention □
Other □

Observation □
Demonstration/modeling □
Video example □

Reflection questions:

Overall, what worked well during the visit?

What do you see as areas for improvement/needs?

Is there any support you need in supervision based on this visit?

Cultural and program adaptations of the Snapshot form

**Original Snapshot form***	**Swedish version**
Home visitor name	Omitted
Date of visit	Included
Date of Snapshot form completion	Included
Duration of visit	Included
When did the family enroll?	Omitted
Age of youngest child in family	Included (age of first child/first child born in Sweden)
Prior to this visit, did you have identified goals for this family?	Omitted
Describe the preparation that occurred prior to this visit	Omitted
How did you select the content you planned to cover during this visit?	Omitted
Interpreter present	Added
Visit location	Added
Professionals present	Added
Content covered (18 topics); 2 response options	Content delivered (8 topics plus "other"); Adapted to program features.<br>Addition of a third response option
Methods used to select visit content	Omitted
Provider patient relationship quality and quality of home visit (5 response options each)	Changed to Likert scale 1-10 along 3 dimensions to rate in each category
Consistently assess family strengths and needs	This is a core program practice, therefore was included in self-efficacy measure
Use of progress monitoring and assessment (of families)	Omitted. Measuring progress is not a program objective. Instead, one question was added about whether professionals assessed parent satisfaction (in any way) following the visit.
Referrals to expand program outreach and effectiveness (9 options plus other) + follow up	12 options + follow-up, adapted to program goals and local context
Referral follow up	Registration of 6 indicators of family participation in community services based on program goals and context
Actions taken during the home visit (11 plus other)	8 plus "other" adapted to program & context
(Separate measure of competency and self-efficacy)	Self-efficacy: 7-point Likert rating scale measuring degree of satisfaction with own abilities. Adapted to core program practices

* Schachner A, Gaylor E, Chen W-B, Hudson L, Garcia D. RISE Home Visiting Evaluation: Final Evaluation Report. Selected findings from years 1 and 4 of the evaluation. SRI International, 2017.
